# Incidence, Microbiological Studies, and Factors Associated With Prosthetic Joint Infection After Total Knee Arthroplasty

**DOI:** 10.1001/jamanetworkopen.2023.40457

**Published:** 2023-10-31

**Authors:** Erica J. Weinstein, Alisa J. Stephens-Shields, Craig W. Newcomb, Randi Silibovsky, Charles L. Nelson, Judith A. O’Donnell, Laurel J. Glaser, Evelyn Hsieh, Jennifer S. Hanberg, Janet P. Tate, Kathleen M. Akgün, Joseph T. King, Vincent Lo Re

**Affiliations:** 1 Division of Infectious Diseases, Department of Medicine, Perelman School of Medicine, University of Pennsylvania, Philadelphia; 2 Center for Real-World Effectiveness and Safety of Therapeutics, Center for Clinical Epidemiology and Biostatistics, Department of Biostatistics, Epidemiology, and Informatics, Perelman School of Medicine, University of Pennsylvania, Philadelphia; 3 Department of Orthopedic Surgery, Perelman School of Medicine, University of Pennsylvania, Philadelphia; 4 Department of Pathology, Perelman School of Medicine, University of Pennsylvania, Philadelphia; 5 Veterans Affairs (VA) Connecticut Health System, West Haven; 6 Section of Rheumatology, Allergy and Immunology, Department of Medicine, Yale University School of Medicine, New Haven, Connecticut; 7 Department of Medicine, Massachusetts General Hospital, Boston; 8 Department of Medicine, Yale University School of Medicine, New Haven, Connecticut; 9 Section of Pulmonary, Critical Care, and Sleep Medicine, VA Connecticut Health System, West Haven; 10 Section of Pulmonary, Critical Care, and Sleep Medicine, Yale University School of Medicine, New Haven, Connecticut; 11 Department of Neurosurgery, Yale University School of Medicine, New Haven, Connecticut

## Abstract

**Importance:**

Despite the frequency of total knee arthroplasty (TKA) and clinical implications of prosthetic joint infections (PJIs), knowledge gaps remain concerning the incidence, microbiological study results, and factors associated with these infections.

**Objectives:**

To identify the incidence rates, organisms isolated from microbiological studies, and patient and surgical factors of PJI occurring early, delayed, and late after primary TKA.

**Design, Setting, and Participants:**

This cohort study obtained data from the US Department of Veterans Affairs (VA) Corporate Data Warehouse on patients who underwent elective primary TKA in the VA system between October 1, 1999, and September 30, 2019, and had at least 1 year of care in the VA prior to TKA. Patients who met these criteria were included in the overall cohort, and patients with linked Veterans Affairs Surgical Quality Improvement Program (VASQIP) data composed the VASQIP cohort. Data were analyzed between December 9, 2021, and September 18, 2023.

**Exposures:**

Primary TKA as well as demographic, clinical, and perioperative factors.

**Main Outcomes and Measures:**

Incident hospitalization with early, delayed, or late PJI. Incidence rate (events per 10 000 person-months) was measured in 3 postoperative periods: early (≤3 months), delayed (between >3 and ≤12 months), and late (>12 months). Unadjusted Poisson regression was used to estimate incidence rate ratios (IRRs) with 95% CIs of early and delayed PJI compared with late PJI. The frequency of organisms isolated from synovial or operative tissue culture results of PJIs during each postoperative period was identified. A piecewise exponential parametric survival model was used to estimate IRRs with 95% CIs associated with demographic and clinical factors in each postoperative period.

**Results:**

The 79 367 patients (median (IQR) age of 65 (60-71) years) in the overall cohort who underwent primary TKA included 75 274 males (94.8%). A total of 1599 PJIs (2.0%) were identified. The incidence rate of PJI was higher in the early (26.8 [95% CI, 24.8-29.0] events per 10 000 person-months; IRR, 20.7 [95% CI, 18.5-23.1]) and delayed periods (5.4 [95% CI, 4.9-6.0] events per 10 000 person-months; IRR, 4.2 [95% CI, 3.7-4.8]) vs the late postoperative period (1.3 events per 10 000 person-months). *Staphylococcus aureus* was the most common organism isolated overall (489 [33.2%]); however, gram-negative infections were isolated in 15.4% (86) of early PJIs. In multivariable analyses, hepatitis C virus infection, peripheral artery disease, and autoimmune inflammatory arthritis were associated with PJI across all postoperative periods. Diabetes, chronic kidney disease, and obesity (body mass index of ≥30) were not associated factors. Other period-specific factors were identified.

**Conclusions and Relevance:**

This cohort study found that incidence rates of PJIs were higher in the early and delayed vs late post-TKA period; there were differences in microbiological cultures and factors associated with each postoperative period. These findings have implications for postoperative antibiotic use, stratification of PJI risk according to postoperative time, and PJI risk factor modification.

## Key Points

**Question:**

What are the incidence rates, microbiological culture results, and factors associated with prosthetic joint infection (PJI) at different periods after primary total knee arthroplasty (TKA)?

**Findings:**

In this cohort study of 79 367 veterans who underwent primary TKA in the US Department of Veterans Affairs health system, the incidence of PJI was highest in the first 3 months and remained elevated through 12 months compared with 12 months or more after surgery. Gram-negative organisms were more prevalent in early vs delayed or late PJIs, and differences in factors of PJI were identified for each postoperative period.

**Meaning:**

Findings of this study suggest that empirical gram-negative antibiotic therapy should be considered for early PJI after primary TKA, and the findings have implications for postoperative antibiotic use, stratification of PJI risk according to postoperative time, and PJI risk factor modification.

## Teaser

This cohort study examines incidents rates, microbiological studies, and patient and surgical factors associated with prosthetic joint infection at 3 time points after total knee arthroplasty among US veterans.

## Introduction

Total knee arthroplasty (TKA) is one of the most common elective surgeries in the US due to increasing rates of obesity and an aging population.^1,2^ Prosthetic joint infection (PJI), one of the most serious complications of TKA, can result in substantial morbidity, mortality, and health care costs.^3^ Despite the clinical implications of PJIs, there are major knowledge gaps regarding the epidemiological features, microbiological studies, and factors of these infections in the US.

Previous studies of PJI after primary (ie, initial) TKA conducted predominantly with claims data were limited by unvalidated or surrogate end points,^4,5,6,7,8^ had short follow-up duration,^9,10^ focused on adults 65 years or older,^7,11,12,13^ involved single centers,^14,15,16,17^ and evaluated few risk factors in mainly unadjusted analyses.^18,19,20,21^ To our knowledge, no US study has compared the incidence, microbiological studies, and factors of PJI occurring in key periods after TKA: early PJI (≤3 months), delayed PJI (between >3 and ≤12 months), and late PJI (>12 months). Prior literature has suggested that the pathogenesis of PJI differs by time points after TKA and that these specified periods may classify the different mechanisms and organisms associated with this infection.^22,23^ These data could identify subgroups at highest risk for PJI after primary TKA and could inform empirical antibiotic therapy for suspected PJI after this surgery.

To address these knowledge gaps, we evaluated a national cohort of veterans who underwent primary TKA within the US Department of Veterans Affairs (VA) Healthcare System. We aimed to identify the incidence rates, organisms isolated from microbiological cultures, and patient and surgical factors of PJI occurring early, delayed, and late after primary TKA.

## Methods

### Study Design and Data Source

We conducted a retrospective cohort study of patients who underwent primary TKA in the national VA system. The Human Investigations Committee at the VA Connecticut Health System, Yale University, and University of Pennsylvania approved the study and waived the informed consent requirement because this research could not practicably be conducted without this waiver. We followed the Strengthening the Reporting of Observational Studies in Epidemiology (STROBE) reporting guideline.^24^

We collected electronic health record (EHR) data from the VA Corporate Data Warehouse. The data set included demographic characteristics, hospital and ambulatory diagnoses (recorded using *International Classification of Diseases, Ninth Revision* [*ICD-9*] and *International Statistical Classification of Diseases and Related Health Problems, Tenth Revision* [*ICD-10*] diagnosis codes), procedures (recorded using *Current Procedural Terminology* codes), laboratory results, microbiological culture results, and dispensed medications. Death date was ascertained from the VA Vital Status File.

To obtain operative variables, we linked patients’ records to the Veterans Affairs Surgical Quality Improvement Program (VASQIP), which included surgical data entered by nurses who reviewed records using standardized definitions.^25^ In general, surgical data at each VA center are identified on an 8-day cycle to ensure that data collection periods begin on different days of the week and are collected for up to 36 surgeries per cycle; thus, not all TKAs are entered into VASQIP. However, exclusion is random and is based on case volume at each center.^26^

### Study Patients

Patients were eligible for inclusion in the study if they (1) underwent elective primary TKA (eTable 1 in Supplement 1) between October 1, 1999, and September 30, 2019, and (2) had at least 1 year of care in the VA prior to TKA. Primary TKAs can be accurately identified from the VA data set with a positive predictive value (PPV) of 95.2% (95% CI, 92%-99%).^27^ Patients were excluded if they underwent partial or revision TKA, underwent TKA for nonelective reasons (malignant neoplasm or fracture), or had a history of prior PJI or native septic joint arthritis of the knee (eTable 1 in Supplement 1).

We defined the index date as the date of hospital admission for primary TKA. The 12 months prior to admission represented the baseline period. Follow-up continued until the occurrence of one of the following: hospitalization with PJI, death, aseptic revision arthroplasty of the knee or contralateral TKA (since these procedures could affect PJI risk), or last VA visit before October 1, 2020 (to ensure patients had the opportunity for at least 1 year of follow-up after TKA).

### Main Study Outcome

The primary end point was incident hospitalization with PJI. We classified patients as being hospitalized with PJI after primary TKA if they had (1) principal or contributory PJI *ICD-9* or *ICD-10* diagnosis codes at hospital discharge; (2) knee radiography within 90 days of PJI diagnosis; and (3) microbiological culture, arthrocentesis, or arthrotomy of the knee within 90 days of PJI diagnosis (eFigure 1 in Supplement 1). This algorithm accurately identified PJI with a PPV of 75.0% (95% CI, 64.1%-84.0%) in the *ICD-9* era and 85.0% (95% CI, 75.3%-92.0%) in the *ICD-10* era.^28^

The PJI date was defined as the date of hospital discharge with PJI diagnosis. We defined early PJI as occurring 3 months or less after primary TKA, delayed PJI as occurring between more than 3 months and 12 months or less after TKA, and late PJI as occurring more than 12 months after TKA. Patients stopped contributing to follow-up at their initial PJI.^29^

### Data Collection

At the time of TKA, we collected data on age, sex, self-reported race and ethnicity, year of surgery, urban vs rural VA center,^30^ body mass index (BMI; calculated as weight in kilograms divided by height in meters squared), and tobacco use (current, former, or never smokers).^31^ Race and ethnicity data were collected as variables to contribute data on the representativeness of the study sample.

Validated algorithms based on 1 or more inpatient or 2 or more outpatient *ICD-9* or *ICD-10* diagnosis codes were used to identify baseline comorbidities, including alcohol use disorder (AUD),^32^ diabetes,^33^ heart failure,^34,35^ hypertension,^36^ hepatitis B virus infection, hepatitis C virus infection,^37^ HIV infection,^38^ and peripheral artery disease (PAD)^39,40^ (eTable 2 in Supplement 1). Autoimmune inflammatory arthritis was defined by 1 inpatient or 2 outpatient *ICD-9* or *ICD-10* diagnosis codes and required at least 1 rheumatologic clinic visit.^41^ Baseline hemoglobin and serum creatinine levels were collected from dates closest to, but within 365 days prior to TKA. Surgical factors extracted from VASQIP were recorded at the time of surgery and included anesthesia technique (general vs regional), American Society of Anesthesiology (ASA) Physical Status Classification System score for preanesthesia comorbidity (range: 1 [indicating a healthy patient] to 4 [indicating a patient with incapacitating severe disease that is a constant threat to life]),^42^ corticosteroid use within 30 days prior to TKA, intraoperative transfusion of packed red blood cells, and operative time (hours).

Results of microbiological cultures performed in the VA were documented in patients’ medical records as free text. All synovial fluid and tissue culture results from the knee within 90 days prior to PJI diagnosis date were obtained. Results were classified as follows: (1) culture-positive, defined as growth of 1 or more bacteria or fungal species from a culture; (2) culture-negative, defined as no growth of organisms; or (3) not collected or missing fluid or tissue culture. Culture-positive specimens were classified according to a hierarchy (eFigure 2 in Supplement 1).

### Statistical Analysis

Among the overall cohort, we estimated incidence rates (events per 10 000 person-months) with 95% CIs of early, delayed, and late PJI. We used unadjusted Poisson regression to estimate the incidence rate ratio (IRR) with 95% CI of early and delayed PJI compared with late PJI. The standard Kaplan-Meier method with noninformative censoring was used to demonstrate the cumulative incidence of PJI at 3, 12, and 24 months. We measured the frequency of gram-positive, gram-negative, fungal, polymicrobial, and culture-negative PJI within each postoperative period and compared the frequencies of organisms using χ^2^ tests.

For the subgroup of patients with linked VASQIP data (referred to as the VASQIP cohort), we explored demographic, baseline clinical, and perioperative factors of early, delayed, and late PJI. The factors we evaluated included age, sex, urban vs rural center, BMI, AUD, hepatitis B virus infection, hepatitis C virus infection, heart failure, HIV infection, hypertension, PAD, autoimmune inflammatory arthritis, tobacco use, anemia (defined as hemoglobin <12.0 g/dL; to convert to milligrams per deciliter, multiply by 10.0), diabetes, chronic kidney disease (CKD; defined by estimated glomerular filtration rate <60 mL/min/1.73 m^2^, calculated using the Modified Diet in Renal Disease equation^43^), anesthesia technique, ASA score, corticosteroid use within 30 days prior to surgery, intraoperative transfusion of packed red blood cells, and prolonged operative time (≥2 hours). The degree of missing data for variables in the VASQIP cohort was low, with the highest being 2.9% for BMI data. As a result, we implemented a complete case analysis to identify factors for PJI in the VASQIP cohort.

We used a piecewise exponential parametric survival model fit through Poisson regression to estimate adjusted IRRs (with 95% CIs) of early, delayed, and late PJI associated with risk factors. Variables were retained in multivariable models if they were associated with PJI (statistical significance: *P* < .10) in univariable analysis and had statistical significance of *P* < .05 in multivariable analysis. We retained BMI in all models given its clinical importance^44^ and conflicting association with PJI in prior studies,^12^ regardless of statistical significance. We used the *svy* module in Stata to account for clustering by VA center and also adjusted for year of TKA (categorized in 5-year periods from 1999 to 2019).^45^ Data were analyzed between December 9, 2021, and September 18, 2023, using Stata, version 16.1 (StataCorp LLC).

## Results

**Figure 1.**
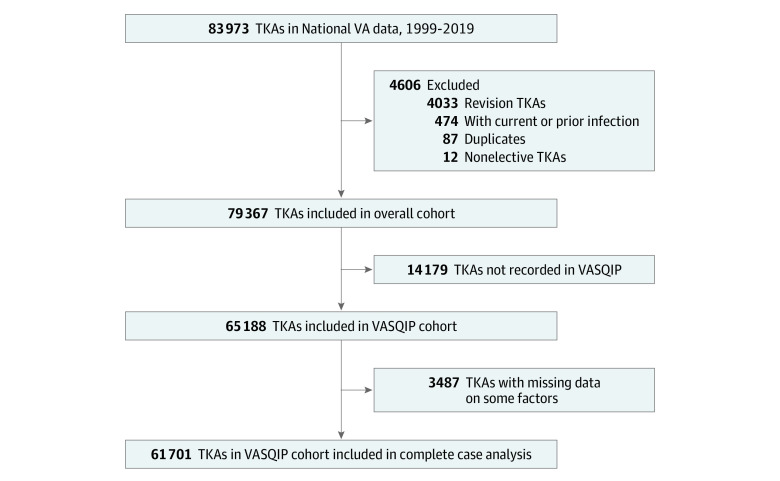
Patients Selected for Inclusion Analyses of incidence rates of early, delayed, and late prosthetic joint infection and microbiological studies were conducted in the overall cohort. Analyses of factors associated with prosthetic joint infection were evaluated in the Veterans Affairs Surgical Quality Improvement Program (VASQIP) complete case cohort. TKA indicates total knee arthroplasty; VA, US Department of Veterans Affairs.

A total of 83 973 patients underwent elective primary TKA during the study period, of whom 4606 were excluded, leaving 79 367 patients in the overall cohort (Figure 1). This cohort comprised 4093 females (5.2%) and 75 274 males (94.8%) with a median (IQR) age of 65 (60-71) years, and most patients identified as non-Hispanic White individuals (73.5%) (Table 1). The median (IQR) duration of follow-up was 58.0 (22.4-108.2) months. Hypertension (86.0%), obesity (BMI of 30.0-39.9 and ≥40; 61.4%), current tobacco use (27.0%), and CKD (18.4%) were common. Among the overall cohort, 82.1% had linked VASQIP data; thus, the VASQIP cohort consisted of 65 188 patients. The characteristics of the VASQIP cohort were similar to those of the overall cohort (eTable 3 in Supplement 1).

**Table 1.** Demographic, Baseline Clinical, and Perioperative Characteristics of the Overall Cohort (n = 79 367)

Characteristic	Patients, No. (%)	Person-months of follow-up
Follow-up, median (IQR), mo	58.0 (22.4-108.2)	5 647 008
Age, y		
Median (IQR)	65 (60-71)	NA
<60	19 682 (24.8)	1 606 579
60-69	34 632 (43.6)	2 394 754
≥70	25 053 (31.6)	1 645 674
Sex		
Female	4093 (5.2)	274 224
Male	75 274 (94.8)	5 372 783
Race and ethnicity^a^		
Hispanic	3484 (4.4)	243 900
Non-Hispanic Black	10 642 (13.4)	734 945
Non-Hispanic White	58 307 (73.5)	4 160 585
Other^b^	4181 (5.3)	328 922
Unknown	2753 (3.5)	178 655
Year of TKA		
1999-2004	12 599 (15.9)	1 503 698
2005-2009	18 767 (23.6)	1 742 641
2010-2014	23 475 (29.6)	1 598 900
2015-2019	24 526 (30.9)	801 767
Urban vs rural center		
Rural	2551 (3.2)	205 065
Urban	76 808 (96.8)	5 441 347
Missing data	8 (<0.1)	NA
BMI		
<25.0	5595 (7.0)	396 666
25.0-29.9	22 114 (27.9)	1 591 410
30.0-39.9	43 315 (54.6)	2 984 255
≥40	5387 (6.8)	412 417
Missing data	2956 (3.7)	NA
Tobacco use		
Never or former	56 851 (71.6)	4 066 693
Current	21 434 (27.0)	1 516 583
Missing data	1082 (1.4)	NA
Baseline comorbidities^c^		
AUD	6436 (8.1)	409 380
Heart failure	3839 (4.9)	220 875
Hypertension	68 054 (86.0)	4 767 230
PAD	3815 (4.8)	232 108
HIV infection	133 (0.2)	9246
HCV infection	2509 (3.2)	188 743
HBV infection	120 (0.2)	9655
Autoimmune inflammatory arthritis	2226 (2.8)	149 622
Anemia	4009 (5.2)	247 181
Missing data	1546 (1.9)	NA
Diabetes	5602 (7.1)	433 175
CKD	14 403 (18.4)	1 018 159
Missing data	1186 (1.5)	NA
Perioperative characteristics obtained from VASQIP cohort^d^		
Corticosteroids use in prior 30 d	1114 (1.7)	72 682
Missing data	1 (<0.1)	NA
ASA score		
1	100 (0.2)	10 098
2	15 314 (23.5)	1 244 416
3	48 000 (73.6)	3 206 147
4	1773 (2.7)	105 895
Missing data	1 (<0.1)	NA
Anesthesia type		
General	41 383 (63.5)	2 980 363
Regional^e^	23 805 (36.5)	1 586 289
Operative time, h		
Median (IQR)	2.0 (1.7-2.5)	
<2	31 030 (47.6)	2 149 297
≥2	34 156 (52.4)	2 417 239
Missing data	2 (<0.1)	NA
Intraoperative transfusion of packed RBCs	359 (0.6)	32 226

Abbreviations: ASA, American Society of Anesthesiologists Physical Status Classification System (score range: 1 [indicating a healthy patient] to 4 [indicating a patient with incapacitating severe disease that is a constant threat to life]); AUD, alcohol use disorder; BMI, body mass index (calculated as weight in kilograms divided by height in meters squared); CKD, chronic kidney disease; eGFR, estimated glomerular filtration rate; HBV, hepatitis B virus; HCV, hepatitis C virus; *ICD-9, International Classification of Diseases, Ninth Revision*; *ICD-10, International Statistical Classification of Diseases and Related Health Problems, Tenth Revision*; NA, not applicable; PAD, peripheral artery disease; RBC, red blood cell; TKA, total knee arthroplasty; VASQIP, Veterans Affairs Surgical Quality Improvement Project.

^a^ Race and ethnicity were self-reported.

^b^ Included Asian, American Indian or Alaska native, Native Hawaiian or Other Pacific Islander, and multiracial.

^c^ Comorbidities of AUD, heart failure, hypertension, diabetes, PAD, HIV infection, HBV infection, and HCV infection were defined by 1 inpatient or 2 outpatient *ICD-9* or *ICD-10* diagnosis codes. Autoimmune inflammatory arthritis was defined as 1 inpatient or 2 outpatient *ICD-9* or *ICD-10* diagnosis codes among those with at least 1 outpatient rheumatologic clinic visit during the baseline period. The *ICD-9* or *ICD-10* diagnosis codes used to identify comorbidities are provided in eTable 2 in Supplement 1. Anemia was defined as hemoglobin level less than 12 g/dL (to convert to grams per liter, multiply by 10.0), and CKD was defined as an eGFR less than 60 mL/min/1.73 m^2^; eGFR was calculated using the Modified Diet in Renal Disease equation: 175 × (serum creatinine)^–1.154^ × (age)^–0.203^ × (0.742, if female) × (1.212, if Black patient).

^d^ Perioperative characteristics were available for only a subset of patients in the VASQIP cohort (n = 65 188). Percentages reflect this subset of patients.

^e^ Included epidural, spinal, or monitored anesthesia.

### Incidence Rates and Microbiological Studies of Early, Delayed, and Late PJIs

**Figure 2.**
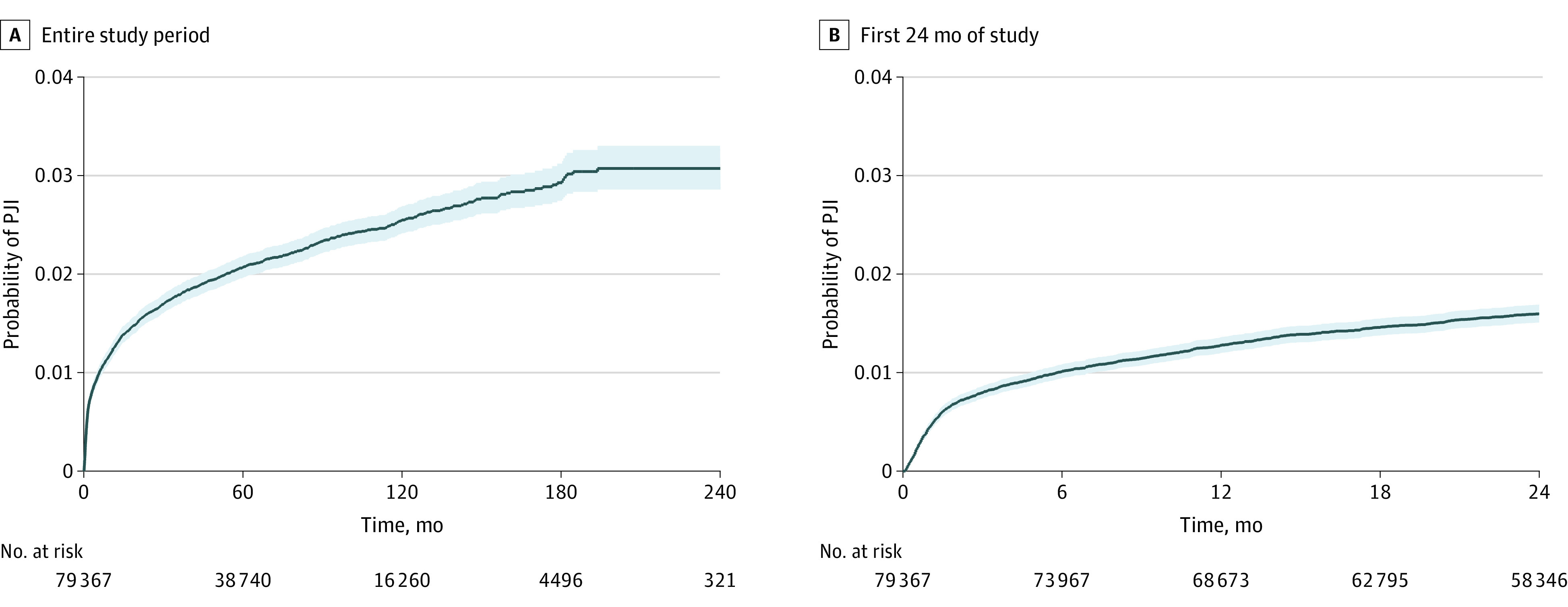
Kaplan-Meier Failure Curves for Prosthetic Joint Infection (PJI) for the Overall Study Period and the First 24 Months After Primary Total Knee Arthroplasty

In the overall cohort, we identified 1599 incident PJIs (2.0%) across all 3 postoperative periods. We observed 627 early PJIs (39.2%) during 234 153 person-months of follow-up, yielding an incidence rate of 26.8 (95% CI, 24.8-29.0) events per 10 000 person-months. We identified 356 delayed PJIs (22.2%) during 654 498 person-months of follow-up, yielding an incidence rate of 5.4 (95% CI, 4.9-6.0) events per 10 000 person-months. There were 616 late PJIs (38.5%) during 4 758 356 person-months of follow-up, yielding an incidence rate of 1.3 (95% CI, 1.2-1.4) events per 10 000 person-months. The rate of PJI after primary TKA was significantly higher in the early (IRR, 20.7; 95% CI, 18.5-23.1) and delayed periods (IRR, 4.2; 95% CI, 3.7-4.8) vs the late period. The cumulative incidence of PJI was 0.80% (95% CI, 0.74%-0.86%) at 3 months, 1.28% (95% CI, 1.21%-1.36%) at 12 months, and 1.60% (95% CI, 1.51%-1.70%) at 24 months (Figure 2).

Among the 1599 incident PJIs, 126 (7.9%) did not have a microbiological culture specimen collected or the culture result was missing. Among 1473 patients with culture results, 459 (31.2%) had culture-negative results (Table 2). A culture-negative result was more common in late PJI compared with the combined early and delayed PJIs (200 [35.1%] vs 259 [28.7%]; *P* = .009). A total of 102 PJIs (6.9%) were polymicrobial, which was more frequently observed in the early postoperative period compared with the combined delayed and late postoperative periods (64 [11.4%] vs 38 [4.2%]; *P* < .001).

**Table 2.** Organisms Isolated From Synovial Fluid or Operative Tissue Cultures of Prosthetic Joint Infections (PJIs) After Primary Total Knee Arthroplasty

Organism	No. (%)			
	Overall (n = 1473)^a^	Early PJI (n = 560)	Delayed PJI (n = 344)	Late PJI (n = 569)
Culture-negative	459 (31.2)	171 (30.5)	88 (25.6)	200 (35.1)
Polymicrobial^b^	102 (6.9)	64 (11.4)	18 (5.2)	20 (3.5)
≥2 Gram-positive	33 (2.4)	20 (3.6)	6 (1.7)	7 (1.2)
≥2 Gram-negative	24 (1.6)	13 (2.3)	9 (2.6)	2 (0.4)
Gram-positive and gram-negative	70 (4.8)	44 (7.9)	10 (2.9)	16 (2.8)
Gram-positive infections	710 (48.2)	286 (51.1)	177 (51.5)	247 (43.4)
*Staphylococcus* species	536 (36.4)	245 (43.8)	123 (35.8)	168 (29.5)
* Staphylococcus aureus*	489 (33.2)	223 (39.8)	108 (31.4)	158 (27.8)
Coagulase-negative staphylococci	286 (19.4)	99 (17.7)	73 (21.2)	114 (20.0)
*Streptococcus* species	122 (8.3)	19 (3.4)	40 (11.6)	63 (11.1)
*Enterococcus* species	65 (4.4)	35 (6.3)	16 (4.7)	14 (2.5)
Anaerobes	10 (0.7)	4 (0.7)	4 (1.2)	2 (0.4)
* Cutibacterium acnes*	6 (0.4)	1 (0.2)	3 (0.9)	2 (0.4)
Other^c^	12 (0.8)	4 (0.7)	1 (0.3)	7 (1.2)
Gram-negative infections	164 (11.1)	86 (15.4)	31 (9.0)	47 (8.3)
* Pseudomonas aeruginosa*	40 (2.7)	19 (3.4)	8 (2.3)	13 (2.3)
* Escherichia coli*	34 (2.3)	19 (3.4)	8 (2.3)	7 (1.2)
*Klebsiella* species	32 (2.2)	18 (3.2)	4 (1.2)	10 (1.8)
*Enterobacter* species	36 (2.4)	23 (4.1)	10 (2.9)	3 (0.5)
*Serratia* species	12 (0.8)	7 (1.3)	2 (0.6)	3 (0.5)
Anaerobes	3 (0.2)	1 (0.2)	1 (0.3)	1 (0.2)
Other^d^	38 (2.6)	16 (2.9)	8 (2.3)	14 (2.5)
Fungal infections^e^	3 (0.2)	0 (0.0)	1 (0.3)	2 (0.4)

^a^ Among the 1599 PJIs in the overall cohort, microbiological data for 126 patients were missing or not collected. Consequently, 1473 PJIs with available microbiological study data were included in these results. Numbers may not sum to group totals or percentages as polymicrobial infections were possible.

^b^ Polymicrobial defined by multiple genera of organisms identified. Numbers may not sum to group totals or percentages as polymicrobial infections with more than 1 organism were possible.

^c^ Included *Micrococcus* species and diphtheroids.

^d^ Included *Salmonella*, *Pasteurella*, *Citrobacter*, *Morganella, Acinetobacter*, *Pasteurella*, *Burkholderia* species, and gram-negative organisms not otherwise specified.

^e^ All fungal infections were identified as *Candida* species.

Gram-positive infections were the most common across all postoperative periods (710 of 1473 infections [48.2%]), with *Staphylococcus aureus* being the most frequently identified (489 [33.2%]). *Staphylococcus* (245 [43.8%]) and *Enterococcus* (35 [6.3%]) species were more common in early PJIs, whereas *Streptococcus* species were more common in delayed (40 [11.6%]) and late (63 [11.1%]) PJIs (Table 2). Gram-negative organisms were observed in 164 infections (11.1%) and more commonly occurred in early PJIs than in combined delayed and late PJIs (86 [15.4%] vs 78 [8.5%]; *P* < .001). *Pseudomonas aeruginosa* was the most common gram-negative organism overall (40 [2.7%]), but *Enterobacter* species were the most frequently isolated in early PJI (23 [4.1%]).

### Risk Factors for Early, Delayed, and Late PJI

Among the 65 188 patients in the VASQIP cohort, 61 701 (94.6%) had complete data on risk factors and so were included in the complete case analysis. Hepatitis C virus infection, PAD, and autoimmune inflammatory arthritis were associated with PJI across all postoperative periods (Table 3). Early PJI was also associated with current smoking, heart failure, hypertension, urban location, and prolonged operative time (≥2 hours). Autoimmune inflammatory arthritis was a robust factor of early PJI (adjusted IRR, 2.3; 95% CI, 1.5-3.5). Delayed PJI was also associated with BMI of 40 or higher, AUD, general anesthesia, anemia, and prolonged operative time (≥2 hours). Body mass index of 40 or higher was a robust factor of delayed PJI (adjusted IRR, 2.7; 95% CI, 1.5-4.9). Late PJI was also associated with AUD, heart failure, anemia, and younger age (<70 years). Anemia was a factor of late PJI (adjusted IRR, 2.2; 95% CI, 1.5-3.1). Factors asociated with PJI at any time after TKA are reported in eTable 4 in Supplement 1. Diabetes and chronic kidney disease were not associated factors.

**Table 3.** Adjusted Incidence Rate Ratios (IRRs) of Prosthetic Joint Infections (PJIs) After Primary Total Knee Arthroplasty (TKA) in the Veterans Affairs Surgical Quality Improvement Project Complete Case Cohort (n = 61 701)

Characteristic	Adjusted IRR (95% CI)^a^		
	Early PJI: ≤3 mo	Delayed PJI: >3 and ≤12 mo	Late PJI: >12 mo
No. of PJIs	513	286	500
Age, y			
<60	1 [Reference]	1 [Reference]	1 [Reference]
60-69	0.9 (0.7-1.1)	0.8 (0.6-1.1)	0.9 (0.7-1.2)
≥70	0.8 (0.6-1.0)	0.8 (0.6-1.2)	0.6 (0.5-0.9)
Urban vs rural center			
Rural	1 [Reference]	1 [Reference]	1 [Reference]
Urban	2.3 (1.1-4.9)	1.3 (0.7-2.6)	1.6 (0.8-3.3)
BMI			
<25.0	1 [Reference]	1 [Reference]	1 [Reference]
25.0-29.9	0.8 (0.6-1.2)	1.7 (0.9-2.9)	0.8 (0.6-1.2)
30.0-39.9	0.8 (0.6-1.2)	1.7 (1.0-2.7)	0.9 (0.7-1.3)
≥40	0.9 (0.6-1.4)	2.7 (1.5-4.9)	1.3 (0.8-1.9)
AUD			
No	1 [Reference]	1 [Reference]	1 [Reference]
Yes	1.1 (0.8-1.6)	2.0 (1.4-2.9)	1.5 (1.1-2.0)
Tobacco use			
Former or never	1 [Reference]	1 [Reference]	1 [Reference]
Current	1.3 (1.0-1.6)	0.9 (0.7-1.2)	1.0 (0.8-1.3)
Heart failure			
No	1 [Reference]	1 [Reference]	1 [Reference]
Yes	1.8 (1.3-2.4)	0.8 (0.5-1.3)	1.9 (1.3-2.8)
Hypertension			
No	1 [Reference]	1 [Reference]	1 [Reference]
Yes	1.5 (1.2-2.0)	1.2 (0.8-1.7)	1.1 (0.8-1.4)
PAD			
No	1 [Reference]	1 [Reference]	1 [Reference]
Yes	1.6 (1.1-2.2)	1.6 (1.0-2.4)	1.9 (1.3-2.7)
HCV infection			
No	1 [Reference]	1 [Reference]	1 [Reference]
Yes	1.9 (1.3-2.8)	1.7 (1.0-2.9)	2.1 (1.5-3.0)
Autoimmune inflammatory arthritis			
No	1 [Reference]	1 [Reference]	1 [Reference]
Yes	2.3 (1.5-3.5)	1.7 (1.0-2.9)	1.9 (1.2-3.0)
Anemia			
No	1 [Reference]	1 [Reference]	1 [Reference]
Yes	1.4 (0.9-1.9)	1.8 (1.3-2.7)	2.2 (1.5-3.1)
Anesthesia type			
Regional	1 [Reference]	1 [Reference]	1 [Reference]
General	1.1 (0.9-1.4)	1.3 (1.0-1.6)	1.1 (0.8-1.3)
Operative time, h			
<2	1 [Reference]	1 [Reference]	1 [Reference]
≥2	1.3 (1.0-1.6)	1.3 (1.0-1.7)	1.1 (0.9-1.4)

Abbreviations: AUD, alcohol use disorder; BMI, body mass index (calculated as weight in kilograms divided by height in meters squared); HCV, hepatitis C virus; PAD, peripheral artery disease; VA, US Department of Veterans Affairs.

^a^ The IRR for each variable was adjusted for all other variables as well as by VA center and year of TKA (categorized in 5-year periods from 1999 to 2019).

## Discussion

Using EHR data from the national VA system, we found that PJI was uncommon after TKA (2.0%), and the incidence rate was highest in the early and delayed periods (20.7 and 4.2 times higher, respectively) compared with the late period. *Staphylococcus* and *Enterococcus* species were more commonly isolated from early PJIs, whereas *Streptococcus* species were more commonly isolated from delayed or late PJIs. Gram-negative infections were more common in the early postoperative period. Hepatitis C infection, PAD, and autoimmune inflammatory arthritis were associated with PJI across all postoperative periods.

This study demonstrated that the rate of PJI was highest within the initial 3 months after primary TKA, and the cumulative incidence at 24 months was 1.60%. These results are consistent with the findings of a cohort study of 69 663 US Medicare beneficiaries in which the cumulative incidence of PJI diagnoses was 1.55% within the first 24 months after elective TKA.^7^ Since the incidence of PJI was highest soon after primary TKA, development and testing of interventions to modify risk factors for early PJI should be prioritized.

Few studies have examined the microbiological characteristics of PJI after TKA. In a cross-sectional study of 1651 patients who had a primary or revision TKA or total hip arthroplasty at Mayo Clinic (2010-2019) and who were observed for PJI, coagulase-negative staphylococci were the most common organisms isolated from cultures.^46^ In a separate cohort study of 231 patients with hip or knee PJI from a single hospital in China (2006-2015), coagulase-negative staphylococci were also the most common organisms isolated.^47^ The finding of the present study that *Staphylococcus aureus* was the most frequently identified causative organism may be due to inclusion of only primary TKAs, whereas previous studies included PJIs of the knee and hip as well as primary and revision arthroplasties.

Gram-negative organisms were isolated from 11.1% of all PJIs and 15.4% of early PJIs. The gram-negative organisms identified from the PJIs in this study, including the early infections, are typically resistant to first-generation cephalosporins. Given these findings, empirical gram-negative antibiotic therapy, in addition to the usual gram-positive coverage, should be considered during suspected early PJI. Cefazolin, the recommended antibiotic for perioperative TKA prophylaxis, would not provide adequate protection against most of the gram-negative organisms identified in this study.^48^ To date, the only intervention associated with a decreased rate of PJI is perioperative antibiotic prophylaxis.^49,50^ Future studies should examine the antimicrobial susceptibility profiles of gram-negative infections that were isolated during early PJI to determine the extent to which broader perioperative gram-negative antibiotic prophylaxis is warranted.

We explored factors of early, delayed, and late PJI. Factors associated with inflammation and tissue hypoxia were associated with PJI in each postoperative period. Hepatitis C virus infection and autoimmune inflammatory arthritis were associated with PJI across all postoperative periods and contributed to systemic inflammation that compromised wound healing.^51,52,53^ Peripheral artery disease was also a significant factor in tissue hypoxia and compromised skin or soft tissue integrity^54^ across the 3 postoperative periods. Early PJI was associated with factors that promoted tissue hypoxia (smoking, heart failure, prolonged operative time, and hypertension).^20,55,56,57,58^ Delayed PJI was associated with factors contributing to tissue hypoxia (prolonged operative time, general anesthesia, and anemia) and impaired wound healing and falls (morbid obesity and AUD).^58,59,60,61,62,63^ Additional factors of late PJI were associated with impaired wound healing and falls (AUD, heart failure, and anemia).^56,59,60,61,62^ Prognostic models should be developed to classify patients according to risk of PJI, which could help guide informed consent, surgical planning, and postoperative monitoring.

We observed a reduced rate of late infections among patients 70 years or older compared with those younger than 60 years, which is consistent with findings of large European and Canadian studies and may reflect a tendency to avoid invasive diagnostic testing and workup for painful joints in patients 70 years or older.^17,64^ Chronic kidney disease and diabetes were not associated with PJI during any period after TKA. The lack of association between CKD and PJI has been reported in a meta-analysis.^65^ Diabetes has been a factor of PJI in most prior studies.^15,16,17,66^ The lack of a similar association in the present study might be due to the definition of diabetes used. No pattern was observed between BMI and PJI risk, making the clinical significance of the association between BMI of 40 or higher and risk of PJI in the delayed postoperative period unclear. The role that obesity plays in postoperative infection risk has been inconsistent, with some studies reporting no association^2,14,21,67^ and other studies observing an increased risk.^12,17,68^ Results of the present study suggest that patients with obesity or CKD should not be restricted from undergoing TKA because of concerns about increased PJI risk.

### Strengths and Limitations

Strengths of this study included the large sample of veterans undergoing primary TKAs, access to national EHR data that included microbiological culture results, and linkage to surgical-specific data. This study also had several potential limitations. First, misclassification of outcomes was possible, but use of a validated PJI end point minimized this risk. It is possible that some PJI outcomes were missed in patients who presented to a non-VA hospital. Second, some culture results could have included organisms that were contaminants. Third, residual confounding by unmeasured factors, such as perioperative antibiotic prophylaxis and chronic skin conditions, could have affected risk factor analyses. Moreover, we evaluated comorbidities as binary (present or absent), without accounting for illness severity, and accounted only for baseline comorbidities or surgical characteristics without evaluating time-varying factors. Fourth, the sample consisted of mostly males with a high prevalence of comorbid conditions. Results may have less generalizability to females and the overall population receiving TKAs.

## Conclusions

In this cohort study of patients who underwent primary TKA in the VA system, incidence rates of PJI occurring in the early and delayed post-TKA periods were higher than incidence of PJI occurring later. *Staphylococcus* and *Enterococcus* species were more commonly isolated from PJIs occurring in the early postoperative period, whereas *Streptococcus* species were more commonly isolated from PJIs occurring in the delayed or late postoperative periods. Gram-negative infections were more commonly observed in the early postoperative period, which suggests that empirical gram-negative antibiotic therapy should be considered along with the usual gram-positive coverage for early PJIs. Additionally, differences in demographic, clinical, and perioperative factors associated with early, delayed, and late PJIs were identified. These findings have implications for postoperative antibiotic use, stratification of PJI risk according to post-TKA periods, and PJI risk factor modification.

## Notes

Supplement 1. **eTable 1.** Diagnosis and Procedures Codes Used to Define Inclusion and Exclusion Criteria, Prosthetic Joint Infection Outcomes, and Censoring Events **eFigure 1.** Validated Case-Finding Algorithms Used to Identify Prosthetic Joint Infection After Total Knee Arthroplasty Within United States Veterans Health Administration Data **eTable 2.** Definitions of Medical Comorbidities and Surgical Characteristics as well as ICD-9 and ICD-10 Diagnosis Codes Used to Identify Comorbidities of Interest **eFigure 2.** Hierarchy Used for Organization of Microbiological Culture Data **eTable 3.** Baseline Demographics, Medical Comorbidities, and Surgical Characteristics at Time of Total Knee Arthroplasty Among Those Not in the Veterans Affairs Surgical Quality Improvement Program Compared to Patients Included in This Cohort **eTable 4.** Adjusted Incidence Rate Ratios of Prosthetic Joint Infection at Any Time After Primary Total Knee Arthroplasty Associated With Demographic, Baseline Clinical, and Peri-Operative Factors Among Patients Included in the Veterans Affairs Surgical Quality Improvement Project (n = 61 701)

Supplement 2.
Data Sharing Statement
