# Supplementary material for: Incidence, Microbiological Studies, and Factors Associated With Prosthetic Joint Infection After Total Knee Arthroplasty
Source: JAMA Netw Open. 2023 Oct 31;6(10):e2340457. doi: 10.1001/jamanetworkopen.2023.40457 (PMC10618849; doi:10.1001/jamanetworkopen.2023.40457)
Supplement: Supplement 1. — eTable 1. Diagnosis and Procedures Codes Used to Define Inclusion and Exclusion Criteria, Prosthetic Joint Infection Outcomes, and Censoring Events eFigure 1. Validated Case-Finding Algorithms Used to Identify Prosthetic Joint Infection After Total Knee Arthroplasty Within United States Veterans Health Administration Data eTable 2. Definitions of Medical Comorbidities and Surgical Characteristics as well as ICD-9 and ICD-10 Diagnosis Codes Used to Identify Comorbidities of Interest eFigure 2. Hierarchy Used for Organization of Microbiological Culture Data eTable 3. Baseline Demographics, Medical Comorbidities, and Surgical Characteristics at Time of Total Knee Arthroplasty Among Those Not in the Veterans Affairs Surgical Quality Improvement Program Compared to Patients Included in This Cohort eTable 4. Adjusted Incidence Rate Ratios of Prosthetic Joint Infection at Any Time After Primary Total Knee Arthroplasty Associated With Demographic, Baseline Clinical, and Peri-Operative Factors Among Patients Included in the Veterans Affairs Surgical Quality Improvement Project (n = 61 701) [file jamanetwopen-e2340457-s001.pdf]

Supplemental Online Content

Weinstein EJ, Stephens-Shields A, Newcomb CW, et al. Incidence, microbiological studies, and factors associated with prosthetic joint infection after total knee arthroplasty. *JAMA Netw Open*. 2023;6(10):e2340457. doi:10.1001/jamanetworkopen.2023.40457

eTable 1. Diagnosis and Procedures Codes Used to Define Inclusion and Exclusion Criteria, Prosthetic Joint Infection Outcomes, and Censoring Events

eFigure 1. Validated Case-Finding Algorithms Used to Identify Prosthetic Joint Infection After Total Knee Arthroplasty Within United States Veterans Health Administration Data

eTable 2. Definitions of Medical Comorbidities and Surgical Characteristics as well as ICD-9 and ICD-10 Diagnosis Codes Used to Identify Comorbidities of Interest

eFigure 2. Hierarchy Used for Organization of Microbiological Culture Data

eTable 3. Baseline Demographics, Medical Comorbidities, and Surgical Characteristics at Time of Total Knee Arthroplasty Among Those Not in the Veterans Affairs Surgical Quality Improvement Program Compared to Patients Included in This Cohort

eTable 4. Adjusted Incidence Rate Ratios of Prosthetic Joint Infection at Any Time After Primary Total Knee Arthroplasty Associated With Demographic, Baseline Clinical, and Peri-Operative Factors Among Patients Included in the Veterans Affairs Surgical Quality Improvement Project (n=61,701)

This supplemental material has been provided by the authors to give readers additional information about their work.

© 2023 Weinstein EJ et al. *JAMA Network Open*

eTable1. Diagnosis and procedures codes used to define inclusion and exclusion criteria, prosthetic joint infection outcomes, and censoring events.

Code	Code Type	Applicability	Description	Applicable time relative to index TKA (Day 0)
81.54	ICD-9	Inclusion	Total knee replacement	NA
0SRDxxx	ICD-10	Inclusion	Replacement of left knee joint, various components, and materials	NA
0SRCxxx	ICD-10	Inclusion	Replacement of right knee joint, various components, and materials	NA
27437	CPT	Exclusion	Revise kneecap	Day 0 or any time prior
27438	CPT	Exclusion	Partial TKA with or without prosthesis	Day 0 or any time prior
27440	CPT	Exclusion	Partial TKA with or without prosthesis	Day 0 or any time prior
27441	CPT	Exclusion	Partial TKA with or without prosthesis	Day 0 or any time prior
27442	CPT	Exclusion	Partial TKA with or without prosthesis	Day 0 or any time prior
27443	CPT	Exclusion	Partial TKA with or without prosthesis	Day 0 or any time prior
27444	CPT	Exclusion	Partial TKA with or without prosthesis	Day 0 or any time prior
27445	CPT	Exclusion	Partial TKA with or without prosthesis	Day 0 or any time prior
27446	CPT	Exclusion	Partial TKA with or without prosthesis	Day 0 or any time prior
27486	CPT	Exclusion	Revision of total knee arthroplasty, with or without allograft; 1 or more components	Day 0 or any time prior
27487	CPT	Exclusion	Revision of total knee arthroplasty, with or without allograft; 1 or more components	Day 0 or any time prior
27488	CPT	Exclusion	Removal of prosthesis, including total knee prosthesis, methylmethacrylate with or without insertion of spacer, knee	Day 0 or any time prior
27132	CPT	Exclusion	Conversion from hemiarthroplasty	Day 0 or any time prior
80.06	ICD-9	Exclusion	Removal of knee prosthesis	Day 0 or any time prior
81.55	ICD-9	Exclusion	Revision of knee replacement NOS	Day 0 or any time prior
00.80	ICD-9	Exclusion	Revision of knee replacement, total (all components)	Day 0 or any time prior
00.81	ICD-9	Exclusion	Revision of knee replacement, tibial component	Day 0 or any time prior
00.82	ICD-9	Exclusion	Revision of knee replacement, femoral component	Day 0 or any time prior
00.83	ICD-9	Exclusion	Revision of knee replacement, patellar component	Day 0 or any time prior
00.84	ICD-9	Exclusion	Revision of total knee replacement, tibial insert (liner)	Day 0 or any time prior
0SWCxxx	ICD-10	Exclusion	Revision of right knee Joint, various approaches	Day 0 or any time prior
0SWDxxx	ICD-10	Exclusion	Revision of left knee joint, various approaches	Day 0 or any time prior
0SWSxxx	ICD-10	Exclusion	Revision of left knee joint, various approaches	Day 0 or any time prior
0SWTxxx	ICD-10	Exclusion	Revision of right knee Joint, various approaches	Day 0 or any time prior

© 2023 Weinstein EJ et al. *JAMA Network Open*

Code	Code Type	Applicability	Description	Applicable time relative to index TKA (Day 0)
0SWUxxx	ICD-10	Exclusion	Revision of synthetic substitute, left knee joint, femoral surface, various approaches	Day 0 or any time prior
0SWVxxx	ICD-10	Exclusion	Revision of right knee Joint, various approaches	Day 0 or any time prior
0SWWxxx	ICD-10	Exclusion	Revision of left knee joint, various approaches	Day 0 or any time prior
0SPCxxx	ICD-10	Exclusion	Removal of device (spacer, synthetic substitute, or liner) from right knee joint, various approaches	Day 0 or any time prior
0SPDxxx	ICD-10	Exclusion	Removal of device (spacer, synthetic substitute, or liner) from left knee joint, various approaches	Day 0 or any time prior
11981	CPT	Exclusion	Spacer insertion	Day 0 or any time prior
11982	CPT	Exclusion	Spacer removal/removal with re-insertion	Day 0 or any time prior
11983	CPT	Exclusion	Spacer removal/removal with re-insertion	Day 0 or any time prior
996.60	ICD-9	Exclusion	Infection and inflammatory reaction due to unspecified device, implant, and graft	Day 0 or any time prior
996.66	ICD-9	Exclusion	Infection due to internal prosthesis (prosthetic joint infection)	Day 0 or any time prior
996.67	ICD-9	Exclusion	Infection and inflammatory reaction due to other internal orthopedic device, implant, and graft device/implant/graft	Day 0 or any time prior
996.69	ICD-9	Exclusion	Infection and inflammatory reaction due to other internal prosthetic device, implant, and graft	Day 0 or any time prior
711.06	ICD-9	Exclusion	Septic arthritis (native joint) lower leg	Day 0 or any time prior
711.08	ICD-9	Exclusion	Septic arthritis, other specified sites, or multiple sites	Day 0 or any time prior
711.09	ICD-9	Exclusion	Septic arthritis, other specified sites, or multiple sites	Day 0 or any time prior
711.96	ICD-9	Exclusion	Septic arthritis (native joint) lower leg	Day 0 or any time prior
170.7x	ICD-9	Exclusion	Malignant neoplasm of long bones of lower limb	During index hospitalization
170.8x	ICD-9	Exclusion	Malignant neoplasm of bone and articular cartilage	During index hospitalization
170.9x	ICD-9	Exclusion	malignant neoplasm of bone and articular cartilage, site unspecified	During index hospitalization
198.5x	ICD-9	Exclusion	secondary neoplasm of bone and bone marrow	During index hospitalization
733.10	ICD-9	Exclusion	Pathologic fracture, site unspecified	During index hospitalization
733.15	ICD-9	Exclusion	Pathologic fracture of other specified part of femur	During index hospitalization
733.16	ICD-9	Exclusion	Pathologic fracture of tibia or fibula	During index hospitalization
733.19	ICD-9	Exclusion	Pathologic fracture of another specified site	During index hospitalization
821.xx	ICD-9	Exclusion	Fracture of other and unspecified parts of femur	During index hospitalization
822.xx	ICD-9	Exclusion	Fracture of patella	During index hospitalization

© 2023 Weinstein EJ et al. *JAMA Network Open*

Code	Code Type	Applicability	Description	Applicable time relative to index TKA (Day 0)
823.xx	ICD-9	Exclusion	Fracture of tibia and fibula	During index hospitalization
827.xx	ICD-9	Exclusion	Other, multiple, and ill-defined fractures of lower limb	During index hospitalization
828.xx	ICD-9	Exclusion	Multiple fractures involving both lower limbs, lower with upper limb and lower limb(s) with rib(s) and sternum	During index hospitalization
829.xx	ICD-9	Exclusion	Fracture of unspecified bones	During index hospitalization
T84.50xx	ICD-10	Exclusion	Joint or orthopedic prosthesis infection unspecified prosthesis	Day 0 or any time prior
T84.53xx	ICD-10	Exclusion	Infection and inflammatory reaction due to internal right knee prosthesis	Day 0 or any time prior
T84.54xx	ICD-10	Exclusion	Infection and inflammatory reaction due to internal left knee prosthesis	Day 0 or any time prior
T84.59xx	ICD-10	Exclusion	Joint or orthopedic prosthesis infection unspecified prosthesis	Day 0 or any time prior
T84.7xxx	ICD-10	Exclusion	Infection and inflammatory reaction due to other internal orthopedic prosthetic devices, implants, and grafts	Day 0 or any time prior
M00.06x	ICD-10	Exclusion	Staphylococcal arthritis, knee	Day 0 or any time prior
M00.16x	ICD-10	Exclusion	Pneumococcal arthritis, knee	Day 0 or any time prior
M00.26x	ICD-10	Exclusion	Other streptococcal arthritis, knee	Day 0 or any time prior
M00.86x	ICD-10	Exclusion	Arthritis due to other bacteria, knee	Day 0 or any time prior
C40.2x	ICD-10	Exclusion	Malignant neoplasm of long bones of lower limb	During index hospitalization
C40.3x	ICD-10	Exclusion	Malignant neoplasm of short bones of lower limb	During index hospitalization
C40.8x	ICD-10	Exclusion	Malignant neoplasm of overlapping sites of bone and articular cartilage of limb	During index hospitalization
C40.9x	ICD-10	Exclusion	Malignant neoplasm of unspecified bones and articular cartilage of limb	During index hospitalization
C41.9x	ICD-10	Exclusion	Malignant neoplasm of bone and articular cartilage, unspecified	During index hospitalization
C79.5x	ICD-10	Exclusion	Secondary malignant neoplasm of bone and bone marrow	During index hospitalization
M84.40x	ICD-10	Exclusion	Pathologic fracture, unspecified site	During index hospitalization
M84.45x	ICD-10	Exclusion	Pathologic fracture, femur, and pelvis	During index hospitalization
M84.46x	ICD-10	Exclusion	Pathologic fracture, tib/fib	During index hospitalization
M84.48x	ICD-10	Exclusion	Pathologic fracture, other site	During index hospitalization
S82.0x	ICD-10	Exclusion	Fracture of patella	During index hospitalization
S82.1x	ICD-10	Exclusion	Fracture of upper end of tibia	During index hospitalization
S82.2x	ICD-10	Exclusion	Fracture of shaft of tibia	During index hospitalization
S82.3x	ICD-10	Exclusion	Fracture of lower end of tibia	During index hospitalization
S82.4x	ICD-10	Exclusion	Fracture of shaft of fibula	During index hospitalization

© 2023 Weinstein EJ et al. *JAMA Network Open*

Code	Code Type	Applicability	Description	Applicable time relative to index TKA (Day 0)
S82.8x	ICD-10	Exclusion	Other fractures of lower leg	During index hospitalization
S82.9x	ICD-10	Exclusion	Unspecified fracture of lower leg	During index hospitalization
996.60	ICD-9	Outcome (PJI)	Infection and inflammatory reaction due to unspecified device, implant, and graft	After Day 0
996.66	ICD-9	Outcome (PJI)	Infection due to internal prosthesis (prosthetic joint infection)	After Day 0
996.67	ICD-9	Outcome (PJI)	Infection and inflammatory reaction due to other internal orthopedic device, implant, and graft device/implant/graft	After Day 0
996.69	ICD-9	Outcome (PJI)	Infection and inflammatory reaction due to other internal prosthetic device, implant, and graft	After Day 0
T81.42XA	ICD-10	Outcome (PJI)	Infection following a procedure, deep incisional surgical site, initial encounter	After Day 0
T81.43XA	ICD-10	Outcome (PJI)	Infection following a procedure, organ and space surgical site, initial encounter	After Day 0
T84.50xx	ICD-10	Outcome (PJI)	Joint or orthopedic prosthesis infection unspecified prosthesis	After Day 0
T84.53xx	ICD-10	Outcome (PJI)	Infection and inflammatory reaction due to internal right knee prosthesis	After Day 0
T84.54xx	ICD-10	Outcome (PJI)	Infection and inflammatory reaction due to internal left knee prosthesis	After Day 0
T84.59xx	ICD-10	Outcome (PJI)	Joint or orthopedic prosthesis infection unspecified prosthesis	After Day 0
T84.7xxx	ICD-10	Outcome (PJI)	Infection and inflammatory reaction due to other internal orthopedic prosthetic devices, implants, and grafts	After Day 0
T85.79XA	ICD-10	Outcome (PJI)	Infection due to other internal prosthetic devices, implants and grafts, initial encounter	After Day 0
73560	CPT	Outcome (PJI)	X-ray knee 1 or 2 views	+/- 90 days from PJI dx
73562	CPT	Outcome (PJI)	X-ray knee 3 views	+/- 90 days from PJI dx
73564	CPT	Outcome (PJI)	X-ray knee 4 views	+/- 90 days from PJI dx
73565	CPT	Outcome (PJI)	X-ray knee bilateral standing	+/- 90 days from PJI dx
73580	CPT	Outcome (PJI)	X-ray knee arthrography	+/- 90 days from PJI dx
20610	CPT	Outcome (PJI)	Arthrocentesis, aspiration and/or injection; major joint	+/- 90 days from PJI dx
20611	CPT	Outcome (PJI)	Arthrocentesis, aspiration and/or injection; major joint, ultrasound guided	+/- 90 days from PJI dx
27310	CPT	Outcome (PJI)	Arthrotomy, knee, with exploration, drainage, or removal of foreign body (e.g., infection)	+/- 90 days from PJI dx
27330	CPT	Outcome (PJI)	Arthrotomy, knee; with synovial biopsy only	+/- 90 days from PJI dx
27331	CPT	Outcome (PJI)	Arthrotomy, knee; including joint exploration, biopsy, or removal of loose or foreign bodies	+/- 90 days from PJI dx
27334	CPT	Outcome (PJI)	Arthrotomy, with synovectomy, knee; anterior or posterior	+/- 90 days from PJI dx

Code	Code Type	Applicability	Description	Applicable time relative to index TKA (Day 0)
27335	CPT	Outcome (PJI)	Arthrotomy, with synovectomy, knee; anterior and posterior including popliteal area	+/- 90 days from PJI dx
87040	CPT	Outcome (PJI)	Culture, bacterial; blood, with isolation and presumptive identification of isolates	+/- 90 days from PJI dx
87999	CPT	Outcome (PJI)	Unlisted microbiology procedure	+/- 90 days from PJI dx
27437	CPT	Censor	Partial TKA with or without prosthesis	After Day 0
27438	CPT	Censor	Partial TKA with or without prosthesis	After Day 0
27440	CPT	Censor	Partial TKA with or without prosthesis	After Day 0
27441	CPT	Censor	Partial TKA with or without prosthesis	After Day 0
27442	CPT	Censor	Partial TKA with or without prosthesis	After Day 0
27443	CPT	Censor	Partial TKA with or without prosthesis	After Day 0
27444	CPT	Censor	Partial TKA with or without prosthesis	After Day 0
27445	CPT	Censor	Partial TKA with or without prosthesis	After Day 0
27446	CPT	Censor	Partial TKA with or without prosthesis	After Day 0
27447	CPT	Censor	Total knee arthroplasty	After Day 0
27486	CPT	Censor	Revision of total knee arthroplasty, with or without allograft; 1 or more components	After Day 0
27487	CPT	Censor	Revision of total knee arthroplasty, with or without allograft; 1 or more components	After Day 0
27488	CPT	Censor	Removal of prosthesis, including total knee prosthesis, methylmethacrylate with or without insertion of spacer, knee	After Day 0
80.06	ICD-9	Censor	Removal of knee prosthesis	After Day 0
81.54	ICD-9	Censor	Total knee replacement	After Day 0
81.55	ICD-9	Censor	Revision of knee replacement NOS	After Day 0
00.80	ICD-9	Censor	Revision of knee replacement, total (all components)	After Day 0
00.81	ICD-9	Censor	Revision of knee replacement, tibial component	After Day 0
00.82	ICD-9	Censor	Revision of knee replacement, femoral component	After Day 0
00.83	ICD-9	Censor	Revision of knee replacement, patellar component	After Day 0
00.84	ICD-9	Censor	Revision of total knee replacement, tibial insert (liner)	After Day 0
0SRDxxx	ICD-10	Censor	Replacement of left knee joint, various components, and materials	After Day 0
0SRCxxx	ICD-10	Censor	Replacement of right knee joint, various components, and materials	After Day 0
0SRTxxx	ICD-10	Censor	Replacement of right knee joint, various components, and materials	After Day 0
0SRUxxx	ICD-10	Censor	Replacement of left knee joint, various components, and materials	After Day 0
0SRVxxx	ICD-10	Censor	Replacement of right knee joint, various components, and materials	After Day 0

© 2023 Weinstein EJ et al. *JAMA Network Open*

Code	Code Type	Applicability	Description	Applicable time relative to index TKA (Day 0)
0SRWxxx	ICD-10	Censor	Replacement of left knee joint, various components, and materials	After Day 0
0SWCxxx	ICD-10	Censor	Revision of right knee Joint, various approaches	After Day 0
0SWDxxx	ICD-10	Censor	Revision of left knee joint, various approaches	After Day 0
0SWSxxx	ICD-10	Censor	Revision of left knee joint, various approaches	After Day 0
0SWTxxx	ICD-10	Censor	Revision of right knee Joint, various approaches	After Day 0
0SWVxxx	ICD-10	Censor	Revision of right knee Joint, various approaches	After Day 0
0SWWxxx	ICD-10	Censor	Revision of left knee joint, various approaches	After Day 0
0SPCxxx	ICD-10	Censor	Removal of device (spacer, synthetic substitute, or liner) from right knee joint, various approaches	After Day 0
0SPDxxx	ICD-10	Censor	Removal of device (spacer, synthetic substitute, or liner) from left knee joint, various approaches	After Day 0

© 2023 Weinstein EJ et al. *JAMA Network Open*

eFigure 1. Validated case-finding algorithms used to identify prosthetic joint infection after total knee arthroplasty within United States Veterans Health Administration data.

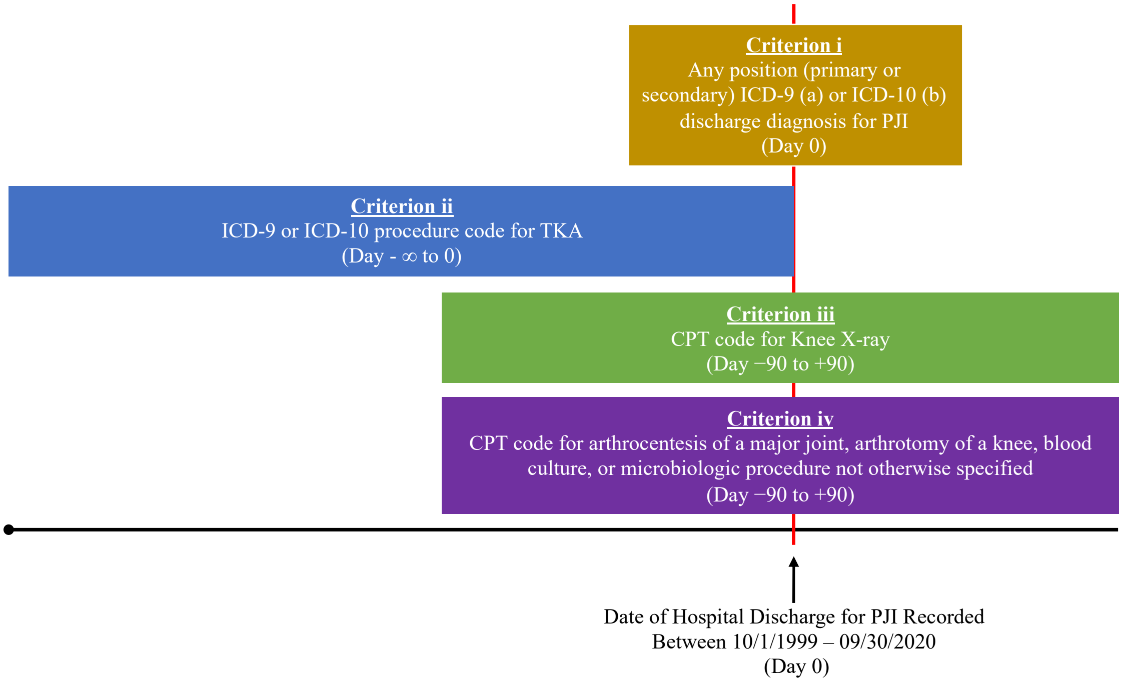

PJI Case-Finding Algorithms: Criteria i(a or b) + ii + iii + iv

Abbreviations: ICD, International Classification of Diseases; PJI, prosthetic joint infection; TKA, total knee arthroplasty. CPT

eTable 2. Definitions of medical comorbidities and surgical characteristics as well as ICD-9 and ICD-10 diagnosis codes used to identify comorbidities of interest.

Alcohol abuse, heart failure, hypertension, diabetes mellitus, peripheral artery disease, HIV infection, HCV infection, HBV infection were defined by the presence of 1 inpatient or 2 outpatient ICD-9/-10 diagnosis codes. Autoimmune inflammatory arthritis was defined as 1 inpatient or 2 outpatient ICD-9/-10 codes among patients with at least one outpatient rheumatologic clinic visit during the baseline period. Anemia was defined as a hemoglobin <12 g/dL. Chronic kidney disease was defined by an estimated glomerular filtration rate (eGFR) of <60 (mL/min/1.73 m²). eGFR was calculated using the Modification of Diet in Renal Disease equation: 175 x (serum creatinine)-1.154 x (age)-0.203 x (0.742, if female) x (1.212, if Black). Steroid use was ascertained from Veterans Affairs Surgical Quality Improvement Program (VASQIP) which documents the regular administration or oral or parenteral steroid use for a chronic condition in the 30-days preoperatively and excludes topical corticosteroids applied to the skin or inhaled or rectal steroids as well as patients receiving pulse-dose steroids only immediately prior to surgery. Anesthesia technique was the principal anesthesia technique utilized for surgery, categorized as general versus regional. Operative time was recorded in minutes, and a prolonged operative time was defined by ≥120 minutes.

Comorbidity	Codes
Alcohol dependence/abuse	ICD-9: 303.00, 303.01, 303.02, 303.03, 303.90, 303.91, 303.92, 303.93, 305.00, 305.01, 305.02, 305.03,<br><br>ICD-10: F10.10, F10.11, F10.120, F10.121, F10.129, F10.130, F10.131, F10.132, F10.139, F10.14, F10.150, F10.151, F10.159, F10.180, F10.181, F10.182, F10.188, F10.19, F10.20, F10.21, F10.220, F10.221, F10.229, F10.230, F10.231, F10.232, F10.239, F10.24, F10.250, F10.251, F10.259, F10.26, F10.27, F10.280, F10.281, F10.282, F10.288, F10.29
Heart failure	ICD-9: 402.01, 402.11, 402.91, 404.01, 404.03, 404.11, 404.13, 404.91, 404.93, 428.XX<br>ICD-10: I09.81, I10.0, I13.0, I25.5, I50.X
Hypertension	ICD-9: 401.0, 401.1, 401.9, 402.00, 402.01, 402.10, 402.11, 402.90, 402.91, 403.0, 403.00, 403.01, 403.1, 403.10, 403.11, 403.9, 403.90, 403.91, 404.0, 404.00, 404.01, 404.02, 404.03, 404.1, 404.10, 404.11, 404.12, 404.13, 404.9, 404.90, 404.91, 404.92, 404.93, 405.01, 405.09, 405.11, 405.19, 405.91, 405.99, 437.2<br><br>ICD-10: I10.X, I11.0, I11.9, I12.0, I12.9, I13.0, I13.10, I13.11, I13.2, I15.0, I15.1, I15.2, I15.8, I15.9, I16.0, I16.1, I16.9, I67.4
Diabetes mellitus	ICD-9: 250.XX, 357.20<br><br>ICD-10: E10.XX, E11.XX, E12.XX, E13.XX, E14.XX, Z46.81, X96.41
Peripheral artery disease	ICD-9: 441.0, 441.00, 441.01, 441.02, 441.03, 441.1, 441.2, 441.3, 441.4, 441.5, 441.6, 441.7, 441.9, 443.81, 443.89, 443.9, 785.4, V43.4<br><br>ICD-10: , I70.0, I70.1, I70.201, I70.202, I70.203, I70.208, I70.209, I70.211, I70.212, I70.213, I70.218, I70.219, I70.221, I70.222, I70.223, I70.228, I70.229, I70.231, I70.232, I70.233, I70.234, I70.235, I70.238, I70.239, I70.241, I70.242, I70.243, I70.244, I70.245, I70.248, I70.249, I70.25, I70.261, I70.262, I70.263, I70.268, I70.269, I70.291, I70.292, I70.293, I70.298, I70.299, I70.301, I70.302, I70.303, I70.308, I70.309, I70.311, I70.312, I70.313, I70.318, I70.319, I70.321, I70.322, I70.323, I70.328, I70.329, I70.331, I70.332, I70.333, I70.334, I70.335, I70.338, I70.339, I70.341, I70.342, I70.343, I70.344, I70.345, I70.348, I70.349, I70.35, I70.361, I70.362, I70.363, I70.368, I70.369, I70.391, I70.392, I70.393, I70.398, I70.399, I70.401, I70.402, I70.403, I70.408, I70.409, I70.411, I70.412, I70.413, I70.418, I70.419, I70.421, I70.422, I70.423, I70.428, I70.429, I70.431, I70.432, I70.433, I70.434, I70.435, I70.438, I70.439, I70.441, I70.442, I70.443, I70.444, I70.445, I70.448, I70.449, I70.45, I70.461, I70.462, I70.463, I70.468, I70.469, I70.491, I70.492, I70.493, I70.498, I70.499, I70.501, I70.502, I70.503, I70.508, I70.509, I70.511, I70.512, I70.513, I70.518, I70.519, I70.521, I70.522, I70.523, I70.528, I70.529, I70.531, I70.532, I70.533, I70.534, I70.535, ICD_DX, I70.538, I70.539, I70.541, I70.542, I70.543, I70.544, I70.545, I70.548, I70.549,

© 2023 Weinstein EJ et al. *JAMA Network Open*

Comorbidity	Codes
	I70.55, I70.561, I70.562, I70.563, I70.568, I70.569, I70.591, I70.592, I70.593, I70.598, I70.599, I70.601, I70.602, I70.603, I70.608, I70.609, I70.611, I70.612, I70.613, I70.618, I70.619, I70.621, I70.622, I70.623, I70.628, I70.629, I70.631, I70.632, I70.633, I70.634, I70.635, I70.638, I70.639, I70.641, I70.642, I70.643, I70.644, I70.645, I70.648, I70.649, I70.65, I70.661, I70.662, I70.663, I70.668, I70.669, I70.691, I70.692, I70.693, I70.698, I70.699, I70.701, I70.702, I70.703, I70.708, I70.709, I70.711, I70.712, I70.713, I70.718, I70.719, I70.721, I70.722, I70.723, I70.728, I70.729, I70.731, I70.732, I70.733, I70.734, I70.735, I70.738, I70.739, I70.741, I70.742, I70.743, I70.744, I70.745, I70.748, I70.749, I70.75, I70.761, I70.762, I70.763, I70.768, I70.769, I70.791, I70.792, I70.793, I70.798, I70.799, I70.8, I70.90, I70.91, I70.92, I71.00, I71.01, I71.02, I71.03, I71.1, I71.2, I71.3, I71.4, I71.5, I71.6, I71.8, I71.9, I73.89, I73.9, I77.1, I79.0, I79.8, K55.1, K55.8, K55.9, Z95.820, Z95.828, Z95.9
Human immunodeficiency virus infection	ICD-9: 042.0, 042.1 042.2, 042.9, V08, , ICD-10: B20
Hepatitis C virus infection	ICD-9: 70.41, 70.44, 70.51, 70.54, 70.70, 70.71, V02.62<br><br>ICD-10: B18.2, B19.20, B19.21, Z22.52
Hepatitis B virus infection	ICD-9: 70.20, 70.21, 70.22, 70.23, 70.30, 70.31, 70.32, 70.33, V02.61<br><br>ICD-10: B18.0, B18.1, B19.1, Z22.51
Autoimmune inflammatory arthritis	ICD-9: 714.X, 696.00, 713.3, 720.00<br><br>ICD-10: M05.X, M06.X, L40.50, L40.52, L40.53, L40.59, M45.X

© 2023 Weinstein EJ et al. *JAMA Network Open*

eFigure 2. Hierarchy used for organization of microbiological culture data.

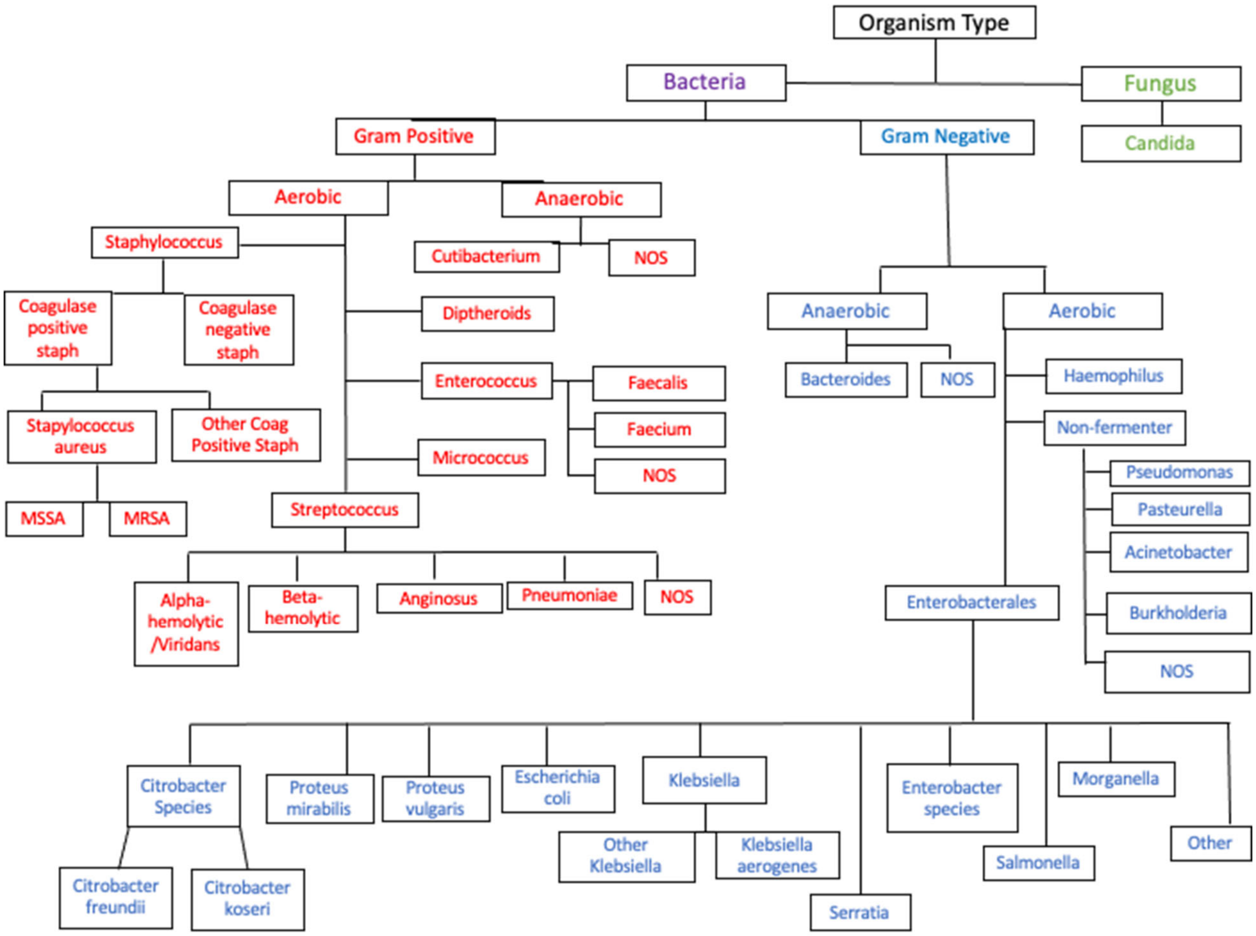

© 2023 Weinstein EJ et al. *JAMA Network Open*

eTable 3. Baseline demographics, medical comorbidities, and surgical characteristics at time of total knee arthroplasty among those not in the Veterans Affairs Surgical Quality Improvement Program compared to patients included in this cohort.

Characteristic[a]	Non-VASQIP (n=14,179) No., %	VASQIP Cohort (n=65,188) No., %
**Follow up in months (median, IQR)**	61.3 (24.4, 114.6)	57.2 (22.0,107.0)
**Age, years**		
Median (IQR)	66.0 (60.0, 72.0)	65.0 (59.0, 71.0)
<60	3,348 (23.6)	16,334 (25.1)
60-69	6,137 (43.3)	28,495 (43.7)
≥70	4,694 (33.1)	20,359 (31.2)
**Sex**		
Male	13,472 (95.0)	61,802 (94.8)
Female	707 (5.0)	3,386 (5.2)
**Race/ethnicity**		
Hispanic	641 (4.5)	2,843 (4.4)
Non-Hispanic Black	1,782 (12.6)	8,860 (13.6)
Non-Hispanic White	10,168(71.7)	48,139 (73.8)
Other[b]	865 (6.0)	3,325 (5.1)
Missing	732 (5.2)	2,021 (3.1)
**Year of TKA Surgery**		
1999-2004	3,410 (24.0)	9,189 (14.1)
2005-2009	2,649 (18.7)	16,118 (24.7)
2010-2014	3,938 (27.8)	19,537 (30.0)
2015-2019	4,182 (29.5)	20,344 (31.2)
**Urban vs rural center**		
Rural	115 (0.8)	2,436 (3.7)
Urban	14,063 (99.2)	62,745 (96.3)
**Body mass index**		
<25.0	1,032 (7.9)	4,563 (7.2)
25.0-29.9	3,943 (30.0)	18,171 (28.7)
30.0-39.9	7,316 (55.8)	35,999 (56.9)
≥40	831 (6.3)	4,556 (7.2)
**Tobacco use**		
Never or former smoker	10,180 (73.5)	46,671 (72.4)
Current smoker	3,678 (26.5)	17,756 (27.6)
**Comorbidities[c]**		
Alcohol dependence/abuse	1,140 (8.1)	5,296 (8.1)
Heart failure	700 (5.0)	3,139 (4.8)
Hypertension	11,953 (84.7)	56,101 (86.3)
Peripheral artery disease	682 (4.8)	3,133 (4.8)
HIV infection	22 (0.2)	111 (0.2)
Hepatitis C virus infection	436 (3.1)	2,073 (3.2)
Hepatitis B virus infection	23 (0.2)	97 (0.1)
Autoimmune inflammatory arthritis	440 (3.1)	1,786 (2.7)
Anemia	779 (5.6)	3,230 (5.0)
Diabetes mellitus	1,285 (9.1)	4,317 (6.6)
Chronic kidney disease	2,530 (18.3)	11,873 (18.4)

Abbreviations:No., number; IQR, interquartile range; TKA, total knee arthroplasty; HIV, Human Immunodeficiency Virus; VASQIP, Veterans Affairs Surgical Quality Improvement Program.

© 2023 Weinstein EJ et al. *JAMA Network Open*

[a]Table only includes characteristics of data available outside of VASQIP

[b] Other race included Asian, American Indian or Alaska native, native Hawaiian or other Pacific Islander, or multiracial.

[c]Anemia was defined as a hemoglobin <12 g/dL. Chronic kidney disease was defined by an estimated glomerular filtration rate (eGFR) of <60 (mL/min/1.73 m²). eGFR was calculated using the Modification of Diet in Renal Disease equation: 175 x (serum creatinine)$^{-1.154}$ x (age)$^{-0.203}$ x (0.742, if female) x (1.212, if Black). Alcohol abuse, heart failure, hypertension, diabetes mellitus, peripheral artery disease, HIV infection, hepatitis B virus infection, and hepatitis C virus infection were defined by 1 inpatient or 2 outpatient ICD-9/-10 diagnosis codes. Autoimmune inflammatory arthritis was defined as 1 inpatient or 2 outpatient ICD-9/-10 diagnosis codes among patients with at least one outpatient rheumatologic clinic visit during the baseline period. ICD-9/10 diagnosis codes used to identify comorbidities are included in **eTable2**

© 2023 Weinstein EJ et al. *JAMA Network Open*

eTable 4. Adjusted incidence rate ratios of prosthetic joint infection at any time after primary total knee arthroplasty associated with demographic, baseline clinical, and peri-operative factors among patients included in the Veterans Affairs Surgical Quality Improvement Project (n=61,701).

Characteristic	Adjusted Incidence Rate Ratio (95% CI)[a]
**Age**	
<60	Ref
60-69	0.9 (0.8-1.0)
≥70	0.8 (0.7-0.9)
**Urban vs rural center**	
Rural	Ref
Urban	1.7 (1.4-2.2)
**Body mass index (kg/m^2)**	
<25.0	Ref
25.0-29.9	0.9 (08-1.2)
30.0-39.9	1.0 (0.8-1.2)
≥40	1.3 (1.0-1.7)
**Alcohol dependence/abuse**	
No	Ref
Yes	1.5 (1.3-1.9)
**Tobacco use**	
Former/never	Ref
Current	1.1 (1.0-1.2)
**Heart failure**	
No	Ref
Yes	1.7 (1.4-2.2)
**Hypertension**	
No	Ref
Yes	1.3 (1.1-1.6)
**Peripheral artery disease**	
No	Ref
Yes	1.8 (1.4-2.2)
**Hepatitis C virus infection**	
No	Ref
Yes	2.0 (1.6-2.5)
**Autoimmune inflammatory arthritis**	
No	Ref
Yes	2.2 (1.7-2.7)
**Anemia**	
No	Ref
Yes	1.8 (1.5-2.2)
**Anesthesia type**	
Regional	Ref
General	1.1 (0.9-1.3)
**Operative time**	
<2 hours	Ref
≥2 hours	1.2 (1.1-1.4)

Abbreviations: Ref, reference; CI, confidence interval.
[a]Incidence rate ratios for each variable were adjusted for all other variables as well as for VA site and year of TKA (categorized in 5-year periods from 1999-2019).

© 2023 Weinstein EJ et al. *JAMA Network Open*
