# Supplementary material for: Incidence, Microbiological Studies, and Factors Associated With Prosthetic Joint Infection After Total Knee Arthroplasty
Source: JAMA Netw Open. 2023 Oct 31;6(10):e2340457. doi: 10.1001/jamanetworkopen.2023.40457 (PMC10618849; doi:10.1001/jamanetworkopen.2023.40457)
Supplement: Supplement 2. — Data Sharing Statement [file jamanetwopen-e2340457-s002.pdf]

Data Sharing Statement

Weinstein. Incidence, Microbiological Studies, and Factors Associated With Prosthetic Joint Infection After Total Knee Arthroplasty. *JAMA Netw Open*. Published October 31, 2023. doi:10.1001/jamanetworkopen.2023.40457

Data
Data available: Yes
Data types: Deidentified participant data
How to access data: Due to US Department of Veterans Affairs (VA) regulations and our ethics agreements, the analytic data sets used for this study are not permitted to leave the VA firewall without a Data Use Agreement. This limitation is consistent with other studies based on VA data. However, VA data are made freely available to researchers with an approved VA study protocol. For more information, please visit https://www.virec.research.va.gov.
When available: With publication

Supporting Documents
Document types: None

Additional Information
Who can access the data: Due to US Department of Veterans Affairs (VA) regulations and our ethics agreements, the analytic data sets used for this study are not permitted to leave the VA firewall without a Data Use Agreement. This limitation is consistent with other studies based on VA data. However, VA data are made freely available to researchers with an approved VA study protocol. For more information, please visit https://www.virec.research.va.gov.
Types of analyses: Due to US Department of Veterans Affairs (VA) regulations and our ethics agreements, the analytic data sets used for this study are not permitted to leave the VA firewall without a Data Use Agreement. This limitation is consistent with other studies based on VA data. However, VA data are made freely available to researchers with an approved VA study protocol. For more information, please visit https://www.virec.research.va.gov.
Mechanisms of data availability: Due to US Department of Veterans Affairs (VA) regulations and our ethics agreements, the analytic data sets used for this study are not permitted to leave the VA firewall without a Data Use Agreement. This limitation is consistent with other studies based on VA data. However, VA data are made freely available to researchers with an approved VA study protocol. For more information, please visit https://www.virec.research.va.gov.
